# Association of psychological symptoms with job burnout and occupational stress among coal miners in Xinjiang, China: A cross-sectional study

**DOI:** 10.3389/fpubh.2022.1049822

**Published:** 2022-12-13

**Authors:** Ailing Fu, Ting Zhao, Xiaoyan Gao, Xinze Li, Xin Liu, Jiwen Liu

**Affiliations:** ^1^Department of Public Health, Xinjiang Medical University, Ürümqi, China; ^2^Department of Medical Record Management, The Affiliated Tumor Hospital of Xinjiang Medical University, Ürümqi, China

**Keywords:** psychological symptoms (PS), job burnout, occupational stress, relationship, coal miners

## Abstract

**Objective:**

The study aimed to investigate the influencing factors of psychological symptoms in relation to job burnout and occupational stress among coal miners in Xinjiang, so as to provide data support for enterprises in an effort to help them identify internal psychological risk factors and improve the mental health of coal miners.

**Methods:**

A cross-sectional study was carried out. A total of 12 coal mines were selected using the stratified cluster random sampling method and 4,109 coal miners were investigated by means of online electronic questionnaires. The Symptoms Check List-90 (SCL-90), Chinese Maslach Burnout Inventory (CMBI), and Job Demand-Control (JDC) model were respectively used to measure the status of psychological symptoms, job burnout, and occupational stress among coal miners. The mediation analysis was performed through structural equation modeling (SEM) by using Analysis of Moment Structure (AMOS).

**Results:**

The prevalence of psychological symptoms was higher in the occupational stress group than in the non-occupational stress group, and increased with job burnout (*P* < 0.05). The multivariate logistic regression analysis results showed that mild (*OR* = 1.401, 95% *CL*: 1.165, 1.685), moderate (*OR* = 2.190, 95% *CL*: 1.795, 2.672), or severe levels of burnout (*OR* = 6.102, 95% *CL*: 3.481, 10.694) and occupational stress (*OR* = 1.462, 95% *CL*: 1.272, 1.679) were risk factors for psychological symptoms in coal miners. The results of structural equation modeling indicated that occupational stress (*β* = 0.11, *P* = 0.002) and job burnout (*β* = 0.46, *P* = 0.002) had significant positive direct effects on psychological symptoms, and job burnout was an intermediate variable between occupational stress and psychological symptoms.

**Conclusion:**

High levels of job burnout and occupational stress were risk factors for psychological symptoms. Both occupational stress and job burnout had direct effects on psychological symptoms, and occupational stress could also have an indirect effect on coal miners' psychological symptoms through the intermediate variable of job burnout.

## Introduction

Psychological disorders posed a serious health problem for the current occupational population (1). The global cost of Psychological disorders is projected to reach US$ 16.3 million by 2030, meaning that these disorders are a major global disease burden (2). The relationship between psychological health and adverse events has been consistently demonstrated. Numerous studies have shown that psychological health were associated with hypertension (3), musculoskeletal disorders (4), and chronic diseases, as well as the occurrence of absenteeism and suicide (5). There was a positive correlation between psychological health and good work outcomes (6).

Psychological disorders were potential factors affecting the quality of life and safe production. Among several self-reported psychopathological assessment tools, the SCL-90 was a popular screening scale for psychopathological testing in clinical populations and was also becoming an established measure of psychiatric symptoms in the general population. The SCL-90 includes an extensive range of psychiatric symptoms from cognition to activity based on factor analysis and can screen for individuals who may have psychiatric symptoms and the type of symptoms (7). The SCL-90 has been the most used instrument to evaluate the psychological health status of Chinese coal miners (8). The occurrence of psychological disorders have been related to a variety of influencing factors, such as the socio-demographic characteristics (e.g., socioeconomic status), occupational characteristics (e.g., work environment, job demands, and control), occupational stress, and job burnout (9, 10). Male general practitioners had poorer mental health than their female counterparts, and middle-aged males were more likely to experience job burnout and psychological disorders (11). Complicated interpersonal relationships, higher job demands, lower job control, and role ambiguity were identified as the main predictors of poor psychological health (12). Studies have also shown that a comprehensive and orderly work environment (13), work rewards, and social support could improve the psychological health level of occupational groups, reducing the risk of psychological disorders (14, 15).

Job burnout was an occupational psychological syndrome that affects safe production (16). The structural theory model was the most widely applied theoretical approach in explaining burnout. The structural theory model viewed burnout as a response to excessive occupational strain, which prompted workers to adopt a series of coping strategies when initially adopted coping strategy failed, which would lead to emotional exhaustion, depersonalization, and reduced fulfillment, which in turn harmed individual and organizational health (17). Job burnout occurred in response to factors such as excessive working hours (18), high job demands, and lower job control (19). Studies have shown that high levels of job burnout were prevalent among nurses (20), psychiatrists (21), and police staff (22), and job burnout was a significant predictor of mortality in the occupational population under 45 years of age (23), and cardiovascular diseases (24). Job burnout contributed to the development of certain mental disorders such as anxiety and depression (23, 25), which has also been identified as an important factor affecting the safety of coal miners (16). Therefore, early identification of symptoms is critical in the work environment, and effective intervention measures should be taken to reduce occupational exhaustion, minimize sources of occupational stress, and prevent the occurrence of depression and anxiety (26).

Occupational stress was a persistent health-impairing physiological and psychological reaction caused by workers' prolonged employment in jobs characterized by high work demands and low work control (27). The JDC model was a widely applied theoretical model of occupational stress that explained the relationship between workers' health and the work environment. The JDC model considered that job demands caused stress and that workers' degree of job control and social support could moderate (or enhance) this stress (28). Heavy workload, long working hours, lower incomes and a poor working environment have been identified as the main reasons giving rise to occupational stress (29). Occupational stress was also positively correlated with the level of criticality, threat, and challenges present in the work environment (30). Occupational stress has persistent negative effects on health with increasing age (31). Associations between occupational stress and coronary heart disease, sleep disturbance (32), negative work performance (33), and suicidal ideation (34) have been reported. Some studies have also shown that long-term high levels of occupational stress may increase the risk of psychological disorders (35), and the relationship between them is a current research hotspot (36).

Cerebrovascular diseases, musculoskeletal diseases, and psychological health problems among coal miners are becoming more prominent as a result of special working environments and occupational stress (37). Psychological health status among coal miners was associated with specific demographic and job characteristics (38). Although coal mining has been automated and mechanized, the working environment of coal miners remains poor (39). These workers were not only exposed to dust and other toxic and harmful chemicals (40), but are also at risk of life-threatening injuries as a result of spontaneous coal combustion (41) and coal mine accidents (39), all of which contribute to occupational stress and job burnout among coal miners.

Previous research often ignored psychological health issues in the study of coal miners' health and the safety of their work environment, even less research has been done on job burnout and psychological health among coal miners, the mediation effect of burnout in occupational stress and psychological symptoms among coal miners in Xinjiang is still a blank. Therefore, this study investigated the psychological health status of coal miners in Xinjiang through a large sample size of data, aimed to explore the relationship between occupational stress, job burnout, and psychological symptoms, and analyze the mediation effect of burnout in occupational stress and psychological symptoms of coal miners. At the same time, this paper lays a foundation for improving the working environment of coal miners, enhancing safety behavior, and promoting coal mine safety production, and also provides data support for enterprises to actively identify internal psychological risk factors.

## Methods

### Participants

This cross-sectional study was conducted from March 2021 to February 2022, and a total of 4,500 coal miners were initially selected by a stratified cluster random sampling method. Our study was conducted with the assistance of the management of coal mines and department managers. Based on the annual output of state-owned coal mines in Xinjiang, the state-owned coal mines in Xinjiang were divided into three categories as follows: small (annual output of fewer than 300,000 tons), medium (300,000–1.2 million tons), and large (1.2–3 million tons). Then, four coal mines were randomly selected from each coal mine category (12 in total), and all of the coal miners from the selected 12 coal mines were taken as the research subjects. Inclusion criteria: (1) all of the coal miners were regular employees who work in the front line of the coal mines and were registered in the coal mining company (excluding administrative managers and logisticians). (2) those who were 18–60 years old and had been employed as a miner for more than 1 year. Exclusion criteria: (1) miners with a history of mental illness. (2) those who did not agree to participate in the questionnaire. (3) questionnaires that were completed in an excessively short amount of time or had more than 20% missing data were excluded, the time taken by the participants to complete the electronic questionnaire could be seen in the background program. 4,261 coal miners who understood the purpose of this study and agreed to participate in the questionnaire.

Finally, a total of 152 questionnaires were excluded as they did not meet the requirements and were incomplete, 4,109 valid questionnaires were retrieved, with an effective recovery rate of 91.31%. According to the sample size calculation formula for cross-sectional study, *N* = $Z_{\alpha }^{2}\times$*pq*/*d*^2^: *N*, Sample size; *Z*_α_, statistics for significance test, *Z*_α_ = 1.96 (significance level α = 0.05); *p*, expected current prevalence, *p* = 37.8% (42), *q* = 1–*p*; *d*, tolerable error, *d* = 0.1*p*; the calculation gives *N* = 632. Taking into account the stratification factor in the sampling method and the questionnaire response rate, we expanded the sample size to three times, *N* = 1,896, our sample size was within the requirements. All participants volunteered to participate in the investigation and provided their written informed consent. This study was approved by the Medical Ethics Committee of the First Affiliated Hospital of Xinjiang Medical University (Approval number: 20170214-174).

### Questionnaire content and research methods

In the cross-sectional study, the questionnaire data were collected by means of the online electronic questionnaires, which were administered using the Questionnaire Star Platform. The online electronic questionnaire was mainly composed of four parts: general demographic information, occupational stress measurement, job burnout measurement and mental health measurement. The detailed content corresponding to the questionnaire can be found below.

### General information investigation

This study collected general information about the coal miners, such as their gender (male or female), age, working years, educational level (high school or below, college degree or above), marital status (unmarried, married, divorced or widowed), shift assignment (day shift, night shift or shifts work), monthly income (<5,000 Yuan or ≥5,000 Yuan), work environment, smoking (yes or no) and alcohol use (yes or no). Smoking was defined as smoking at least one cigarette per day for more than 1 year, and alcohol use was defined as drinking at least twice a week for more than 1 year (43).

### Psychological health investigation

The psychological assessment was carried out using the Chinese version of the Symptoms Check List-90 (SCL-90), which was developed by Derogatis, and was currently recognized as a mental health evaluation scale (4). For the scale in this study, the Cronbach's alpha was 0.986, while the index was 0.987 and the split-half reliability coefficient was 0.94. The scale was scored from 1 to 5 (1 = Not, 2 = Very light, 3 = Medium, 4 = Heavier and 5 = Serious), consisted of 90 items, and contained 10 factors. Each factor did not contain the same number of items; these factors were somatization (12 items), compulsive symptoms (10 items), interpersonal sensitivity (nine items), depression (13 items), anxiety (10 items), hostility (six items) items), phobia (seven items), paranoia (six items), psychosis (10 items), and another (seven items). Psychological symptoms status was assessed on the basis of the SCL-90 total and factor scores. The SCL-90 total score was the total score of all items of the scale; and a higher total score indicated poorer mental health. The factor score was the total score for each factor divided by the number of entries for that factor. The evaluation criteria for psychological symptoms were a total SCL-90 score ≥160, or any factor score >2.

### Job burnout investigation

Job burnout was measured by the Chinese Maslach Burnout Inventory (CMBI), which was compiled by Li Yongxin and had good reliability and validity (44). In this study, the Cronbach's alpha was 0.871, while the KMO index was 0.902 and the split-half reliability coefficient was 0.86. The CMBI scale consisted of 15 items, which included three dimensions: emotional exhaustion (five items), depersonalization (five items) and reduced personal accomplishment (five items). The questionnaire was scored according to a seven-point scale (1 = never, 2 = several times a year, 3 = once a month, 4 = several times a month, 5 = once a week, 6 = several times a week, 7 = every day). Items for emotional exhaustion and depersonalization were forward-scored, while items for reduced personal accomplishment were reverse-scored. The score for each dimension was the total score for each item in that dimension. The critical values for emotional exhaustion, depersonalization, and reduced personal accomplishment were 25, 11, and 16, respectively. According to the critical value of the three dimensions, job burnout was divided into four burnout levels, namely zero burnout (the scores of all three dimensions were less than the critical value), mild burnout (the score of one of the three dimensions was greater than or equal to the critical value; the remaining two were below the critical value), moderate burnout (two of the three dimensions scored greater than or equal to the critical value, and the score of the remaining dimension was below the critical value), and severe burnout (the scores of all three dimensions were greater than or equal to the critical value) (24, 44).

### Occupational stress investigation

The Job Demand-Control (JDC) model was measured by the Chinese version of the Job Content Questionnaire (JCQ) (45). JDC was proposed by Karasek et al. (46), who suggested that occupational stress occurs when workers are exposed to high psychological demands and low job control. The scale has been widely used to study the relationship between work, health, and the wellbeing of workers. For the scale in this study, Cronbach's alpha was 0.895, the KMO index was 0.906 and the split-half reliability coefficient was 0.72. The scale was scored from 1 to 5 (1 = Disagree entirely, 2 = Disagree, 3 = Agree, 4 = Agree strongly, 5 = Agree entirely), consisted of 16 items, which included three dimensions: job demands (five items), job control (six items) and social support (five items). The score of each dimension was the total score of each dimension item divided by the number of items in each dimension. The JDC ratio = job demand/(job control^*^C), where constant C = 5/6. According to the JDC ratio, occupational stress was divided into the occupational stress group (JDC > 1) and the non-occupational stress group (JDC ≤ 1) (45).

### Structural equation model

**Figure 1:**
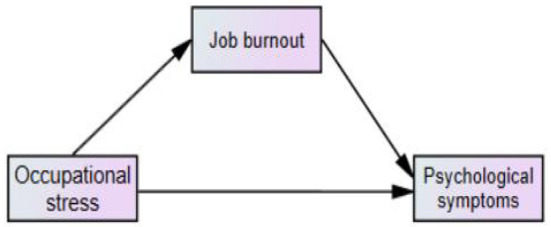
Hypothetical model.

Structural equation modeling (SEM) is a validated hypothesis model, which incorporates factor analysis and path analysis to observe the direct and indirect effects of independent variables on dependent variables and is often used extensively in empirical studies to investigate the relationship between variables (47). Common evaluation indexes for SEM are the Comparative Fit Index (CFI), Goodness of Fit Index (GFI), Adjusted Goodness of Fit Index (AGFI), Tucker-Lewis Index (TLI), and Root Mean Square Error of Approximation (RMSEA), when CFI, GFI, AGFI, TLI are all >0.9 and RMSEA < 0.08 is considered as good model fit (48, 49). Theories were based on the fact that excessive occupational stress was associated with the occurrence of job burnout (17) and mental health (12), and that job burnout also contributed to the prevalence of certain psychological disorders (23, 25), this study established and tested the following hypotheses: occupational stress affects burnout and psychological symptoms, and job burnout has a mediating role in occupational stress and psychological symptoms (Figure 1).

### Quality control

In this study, the electronic version of the questionnaire was used to collect information, and the participants completed the relevant information by scanning the two-dimensional code of the questionnaire. Before distributing the electronic questionnaire to the coal miners, we tested and modified the format many times so that the participants could complete the questionnaire easily. Many items in the questionnaire (e.g., age, gender and marital status) replaced the manual inputting of information with multiple-choice questions, which saved the participants time and improved the response rate of the questionnaire. We also set mandatory field items for some options, which meant that the questionnaire could not be submitted if the mandatory field items had not been completed. If the answers to all of the questions in a questionnaire were the same, this was taken to indicate that the questionnaire may have been completed carelessly. In this event, such questionnaires were discarded.

### Statistical analysis

Statistical analyses were performed by SPSS Windows V. 21.0 (SPSS, Inc., Chicago, IL, USA). Data that did not conform to a normal distribution were statistically described by median (*M*) and interquartile range (*Q R*). For non-normally distributed data, the Mann-Whitney *U* test was used for two-group comparisons, and the Kruskal-Wallis H test was used for multiple-group comparisons. The chi-square test was used to compare categorical variables, while the Wilcoxon rank sum test and trend chi-square test were used to compare rank categorical variables. Multivariate analysis of the factors influencing psychological symptoms was carried out by binary logistic regression analysis. The relationships between occupational stress, burnout, and psychological symptoms were further analyzed by IBM SPSS AMOS version 23.0, and the optimal structural equation model was fitted. The maximum likelihood estimation (MLE) method was used for the parameter estimation of SEM. The significance level was set at 0.05.

## Results

### The prevalence of psychological symptoms among different demographic characteristics of coal miners

A total of 4,109 coal miners participated in this study, 3,875 males (2,083 underground workers and 1,792 open-pit workers) and 234 females (all open pit workers). Among 4,109 coal miners, a total of 1,372 tested positive for psychological symptoms, with a positive detection rate of 33.4%. There were statistically significant differences in the prevalence of psychological symptoms between gender, educational level, marital status, shift assignment, monthly income, work environment, and alcohol use (*P* < 0.05). Compared to other groups of coal miners, the prevalence of psychological symptoms was higher among males (33.8%), participants with a college degree education or above (46.1%), divorced or widowed participants (40.9%), participants with a monthly income of <5,000 Yuan, worked in the underground (38.4%) and participants who used alcohol (34.7 %), although it was lower among those who worked shifts (30.4%) (*P* < 0.05) (Table 1).

**Table 1:** The prevalence of psychological symptoms among different sub-groups (*N* = 4,109).

**Index**	**Groups**	**Total (*N*)**	**Psychological symptoms**		**Chi-squared value**	***P*-value**
			**Number**	**Positive (%)**		
Total	4,109	1,372	33.4	-	-
Gender	Male	3,875	1,308	33.8	4.07	**0.025**
	Female	234	64	27.4		
Age (years)	≤ 30	676	209	30.9	3.04	0.219
	31–45	1,855	616	33.2		
	≥45	1,578	547	34.7		
Educational level	High school or below	3,004	863	28.7	109.15	**< 0.001**
	College degree or above	1,105	509	46.1		
Marital status	Unmarried	501	179	35.7	7.08	**0.029**
	Married	3,415	1,114	32.6		
	Divorced or widowed	193	79	40.9		
Shift assignment	Day shift	1,258	500	39.7	35.08	**< 0.001**
	Night shift	61	24	39.3		
	shifts work	2,790	848	30.4		
Monthly income (Yuan)	< 5,000	759	281	37.0	5.52	**0.011**
	≥5,000	3,350	1,091	32.6		
Work environment	Open-pit	1,980	555	28.0	49.36	**< 0.001**
	Underground	2,129	817	38.4		
Type of coal mines	Large	2,175	708	32.6	1.61	0.448
	Medium	1,362	464	34.1		
	Small	572	200	35.0		
Smoking	No	1,671	535	32.0	2.39	0.065
	Yes	2,438	837	34.3		
Alcohol use	No	1,982	633	31.9	3.63	**0.031**
	Yes	2,127	739	34.7		

The bold values indicate the values of *P* < 0.05.

### Comparison of SCL-90 dimensions score among different sub-groups

There was a significant difference between males and females in scores on interpersonal sensitivity and another factor (*P* < 0.05). Scores on all dimensions except obsessive-compulsive symptoms, interpersonal sensitivity, and paranoia differed statistically among age groups (*P* < 0.05). Among all dimensions, there were significant differences in education level, shift assignment, and work types groups (*P* < 0.001). Scores for somatization and compulsive symptoms were significantly different between marital status and monthly income groups (*P* < 0.05); the scores for all dimensions were significantly higher in the alcohol use group than in the non-alcohol use group (*P* < 0.05), except for compulsive symptoms and another factor. Statistically significant differences (*P* < 0.05) were found between the different smoking groups for obsessive-compulsive symptoms and interpersonal sensitivity (Table 2).

**Table 2:** Comparison of SCL-90 dimensions score among different sub-groups [*M* (*Q R*)].

**Groups**	***N***	**SO**	**CS**	**IS**	**DE**	**AN**	**HO**	**PH**	**PA**	**PS**	**AF**
Gender
Male	3,875	14.0 (9.0)	17.0 (13.0)	13.0 (9.0)	17.0 (12.0)	11.0 (8.0)	7.0 (4.0)	7.0 (3.0)	7.0 (5.0)	12.0 (6.0)	10.0 (4.0)
Female	234	15.0 (9.0)	16.0 (11.0)	11.0 (7.0)	16.0 (10.0)	12.0 (6.0)	7.0 (3.0)	7.0 (3.0)	7.0 (3.0)	11.0 (5.0)	10.0 (6.0)
*Z*-value		−1.593	−0.763	−2.164	−1.499	−0.164	−0.571	−1.008	−0.773	−0.672	−2.269
*P*-value		0.111	0.445	**0.030**	0.134	0.870	0.568	0.314	0.440	0.501	**0.023**
Educational level
High school or below	3,004	14.0 (8.0)	16.0 (11.0)	12.0 (8.0)	16.0 (10.0)	11.0 (6.0)	7.0 (3.0)	7.0 (2.0)	7.0 (4.0)	11.0 (5.0)	10.0 (4.0)
College degree or above	1,105	17.0 (11.5)	20.0 (11.5)	15.0 (10.0)	20.0 (15.0)	13.0 (11.0)	8.0 (6.0)	7.0 (6.0)	8.0 (6.0)	13.0 (10.0)	11.0 (6.0)
*Z*-value		−10.423	−12.218	−11.868	−11.208	−10.524	−10.924	−6.854	−11.664	−10.172	−9.910
*P*-value		**< 0.001**	**< 0.001**	**< 0.001**	**< 0.001**	**< 0.001**	**< 0.001**	**< 0.001**	**< 0.001**	**< 0.001**	**< 0.001**
Marital status
Unmarried	501	14.0 (8.0)	18.0 (13.0)	13.0 (9.0)	16.0 (13.0)	11.0 (8.0)	6.0 (5.0)	7.0 (3.0)	7.0 (5.0)	11.0 (6.5)	10.0 (5.0)
Married	3,415	15.0 (9.0)^a^	17.0 (13.0)	12.0 (9.0)	16.0 (11.0)	12.0 (8.0)	7.0 (4.0)	7.0 (3.0)	7.0 (4.0)	12.0 (6.0)	10.0 (5.0)
Divorced or widowed	193	16.0 (11.0)^a^	20.0 (12.0)^b^	14.0 (11.5)^b^	18.0 (14.0)	12.0 (9.0)	7.0 (5.0)	7.0 (4.0)	8.0 (5.0)	13.0 (8.0)	11.0 (6.0)^b^
Chi-squared value		11.888	8.180	13.157	4.468	2.014	2.010	0.522	4.882	3.826	11.481
*P*-value		**0.003**	**0.017**	**0.001**	0.107	0.365	0.366	0.770	0.087	0.148	**0.003**
Shift assignment
Day shift	1,258	16.0 (10.0)	19.0 (12.0)	14.0 (10.0)	18.0 (13.0)	12.0 (10.0)	8.0 (6.0)	7.0 (4.0)	8.0 (6.0)	12.0 (8.0)	10.0 (5.0)
Night shift	61	15.0 (11.0)	15.0 (14.0)	14.0 (12.0)	17.0 (12.5)	12.0 (9.5)	8.0 (4.0)	7.0 (3.0)	7.0 (4.0)	12.0 (6.5)	10.0 (6.5)
Work shifts	2,790	14.0 (8.0)^c^	16.0 (12.0)^c^	12.0 (9.0)^c^	16.0 (11.0)^c^	11.0 (7.0)^c^	6.0 (4.0)^c^	7.0 (2.0)^c^	7.0 (4.0)^c^	11.0 (6.0)^c^	10.0 (5.0)^c^
Chi-Squared Value		40.838	49.960	46.085	57.120	68.541	69.141	22.076	37.926	44.085	22.178
*P*-value		**< 0.001**	**< 0.001**	**< 0.001**	**< 0.001**	**< 0.001**	**< 0.001**	**< 0.001**	**< 0.001**	**< 0.001**	**< 0.001**
Monthly income (Yuan)
< 5,000	759	15.0 (11.0)	18.0 (13.0)	13.0 (10.0)	17.0 (13.0)	12.0 (10.0)	7.0 (5.0)	7.0 (4.0)	7.0 (5.0)	12.0 (8.0)	10.0 (5.0)
≥5,000	3,350	14.0 (8.0)	17.0 (13.0)	13.0 (9.0)	17.0 (12.0)	11.0 (8.0)	7.0 (4.0)	7.0 (3.0)	7.0 (4.0)	12.0 (6.0)	10.0 (5.0)
*Z*-value		−4.177	−2.472	−0.403	−0.160	−1.845	−0.596	−1.874	−0.556	−2.029	−2.682
*P*-value		**< 0.001**	**0.013**	0.687	0.873	0.065	0.551	0.061	0.578	**0.043**	**0.007**
Work types
Open-pit	13.0 (7.0)	16.0 (12.0)	12.0 (8.0)	15.0 (10.0)	11.0 (6.0)	6.0 (3.0)	7.0 (2.0)	7.0 (3.0)	11.0 (4.0)	10.0 (3.0)
Underground	16.0 (11.0)	18.0 (13.0)	13.0 (10.0)	18.0 (13.0)	13.0 (10.0)	8.0 (6.0)	7.0 (4.0)	8.0 (6.0)	12.0 (8.0)	10.0 (6.0)
Z-value		−13.746	−4.669	−6.798	−9.682	−11.963	−11.544	−9.335	−8.767	−10.565	−1.078
*P*-value		**< 0.001**	**< 0.001**	**< 0.001**	**< 0.001**	**< 0.001**	**< 0.001**	**< 0.001**	**< 0.001**	**< 0.001**	**< 0.001**
Alcohol use
No	1,982	13.0 (7.0)	17.0 (13.0)	12.0 (9.0)	16.0 (11.0)	11.0 (6.0)	6.0 (3.0)	7.0 (2.0)	7.0 (4.0)	11.0 (5.0)	10.0 (4.0)
Yes	2,127	16.0 (10.0)	17.0 (12.0)	13.0 (9.0)	17.0 (13.0)	12.0 (9.0)	8.0 (5.0)	7.0 (4.0)	7.0 (5.0)	12.0 (8.0)	10.0 (6.0)
*Z*-value		−11.672	−0.813	−2.807	−6.812	−9.443	−11.166	−8.719	−7.632	−7.946	−1.709
*P*-value		**< 0.001**	0.416	**0.005**	**< 0.001**	**< 0.001**	**< 0.001**	**< 0.001**	**< 0.001**	**< 0.001**	0.087

SO, Somatization; CS, Compulsive symptoms; IS, Interpersonal sensitivity; DE, Depression; AN, Anxiety; HO, Hostility; PH, Phobia; PA, Paranoia; PS, Psychosis; AF, Another factor.

Compared with the unmarried group, ^a^*P* < 0.05; Compared with the married group, ^b^*P* < 0.05; Compared with the day shift group, ^c^*P* < 0.05. The bold values indicate the values of *P* < 0.05.

### The association between job burnout and psychological symptoms among coal miners

Among 4,109 coal miners, a total of 3,179 coal miners reported different levels of job burnout in the study, accounting for about 77.4%. Among them, 1,965 (47.8%) had mild levels of burnout, 1,146 (27.9%) had moderate levels of burnout, and 68 reported severe levels of burnout (1.7%). The results of the Wilcoxon rank sum test showed that there was a statistically significant difference in the positive detection rate of psychological symptoms among different job burnout groups of coal miners (*T* = −10.49, *P* < 0.001). The results of the trend chi-square test showed that the positive detection rate of psychological symptoms increased in line with higher burnout levels (Chi-squared value = 116.85, *P* < 0.001), which suggested that there was a relationship between job burnout and psychological symptoms among coal miners (Table 3).

**Table 3:** Comparison of psychological symptoms among different job burnout groups.

**Job burnout**	**Total**	**Psychological**		***T*-value**	***P*-value**
**groups**	***N* (%)**	**symptoms**			
		**Number**	**Positive (%)**		
Zero burnout	930 (22.6)	222	23.9	−10.49	**<** **0.001**
Mild burnout	1,965 (47.8)	613	31.2		
Moderate burnout	1,146 (27.9)	489	42.7		
Severe burnout	68 (1.7)	48	70.6		

The bold values indicate the values of *P* < 0.05.

### The association between occupational stress and psychological symptoms among coal miners

In this study, a total of 1,564 coal miners reported that they experienced occupational stress, accounting for 38.1%. The chi-squared test showed that compared with the non-occupational stress group of coal miners, the occupational stress group had a higher positive detection rate of psychological symptoms (χ^2^ = 67.71, *P* < 0.001), which suggested that there was a relationship between occupational stress and psychological symptoms among the coal miners (Table 4).

**Table 4:** Comparison of psychological symptoms between different occupational stress groups.

**Occupational stress groups**	**Total *N* (%)**	**Psychological symptoms**		**Chi-squared value**	***P*-value**
		**Number**	**Positive (%)**		
Non-occupational stress	2,545 (61.9)	729	28.6	67.71	**< 0.001**
Occupational stress	1,564 (38.1)	643	41.1		

The bold values indicate the values of *P* < 0.05.

### Multivariate logistic regression analysis of psychological disorder among coal miners

After setting dummy variables for multiple classification factors, multivariate logistic regression analysis was carried out using the forward stepwise method, i.e., the likelihood ratio method. The presence or absence of a psychological disorder was taken as the dependent variable, while the variables shown in Table 1 with statistical significance, as well as job burnout and occupational stress were the independent variables, and a multivariate analysis of psychological symptoms among coal miners was carried out (the variable assignment was shown in Table 5). The results of the multivariate logistic regression analysis showed that male gender (*OR* = 1.421, 95% *CL*: 1.036, 1.948), college degree education or above (*OR* = 1.921, 95% *CL*: 1.641, 2.249), worked in the underground (*OR* = 1.383, 95% *CL*: 1.199, 1.595), mild (*OR* = 1.401, 95% *CL*: 1.165, 1.685), moderate (*OR* = 2.190, 95% *CL*: 1.795, 2.672), or severe levels of burnout (*OR* = 6.102, 95% *CL*: 3.481, 10.694) and occupational stress (*OR* = 1.462, 95% *CL*: 1.272, 1.679) were risk factors for psychological symptoms in coal miners. However, being unmarried (*OR* = 0.641, 95% *CL*: 0.472, 0.871) or married (*OR* = 0.656, 95% *CL*: 0.459, 0.935), shifts work (*OR* = 0.801, 95% *CL*: 0.687, 0.934) and monthly income ≥5,000 Yuan (*OR* = 0.695, 95% *CL*: 0.581, 0.832) were protective factors against psychological symptoms in coal miners (Table 6).

**Table 5:** Variable assignment: Logistic regression analysis.

**Variable**	**Name of variable**	**Assignment**
Y	Psychological symptoms	0 = No, 1 = Yes
X1	Gender	0 = Female, 1 = Male
X2	Educational level	0 = High school or below, 1 = College degree or above
X3	Marital status	0 = Divorced or widowed, 1 = Unmarried, 2 = Married
X4	Shift assignment	0 = Day shift, 1 = Night shift, 2 = Shifts work
X5	Monthly income (Yuan)	0 = < 5,000, 1 = ≥5,000
X6	Work environment	0 = Open-pit, 1 = Underground
X7	Alcohol use	0 = No, 1 = Yes
X8	Job burnout	0 = Zero burnout, 1=Mild burnout, 2 = Moderate burnout, 3 = Severe burnout
X9	Occupational stress	0 = Non-occupational stress, 1 = Occupational stress

**Table 6:** Multivariate logistic regression analysis of psychological symptoms.

**Variable**	**Comparison group**	**β**	**S. E**	**Wald**	***P*-value**	**Odds ratio (95%CL)**	***VIF***
Constant	−1.104	0.228	23.37	**< 0.001**	0.332 (-, -)	-
Gender	Female (Ref.)	-	-	-	-	-	1.10
	Male	0.351	0.161	4.76	**0.029**	1.421 (1.036, 1.948)	
Educational level	High school or below (Ref.)	-	-	-	**-**	-	1.16
	College degree or above	0.653	0.080	65.83	**< 0.001**	1.921 (1.641, 2.249)	
Marital status	Divorced or widowed (Ref.)	-	-	8.07	**0.018**	-	1.02
	Unmarried	−0.422	0.181	5.42	**0.020**	0.656 (0.459, 0.935)	
	Married	−0.444	0.156	8.07	**0.005**	0.641 (0.472, 0.871)	
Shift assignment	Day shift (Ref.)	-	-	8.82	**0.012**	-	1.13
	Night shift	0.084	0.282	0.09	0.765	1.088 (0.626, 1.89)	
	Shifts work	−0.222	0.078	8.05	**0.005**	0.801 (0.687, 0.934)	
Monthly income /Yuan	< 5,000 (Ref.)	-	-	-	-	-	1.10
	≥5,000	−0.364	0.091	15.80	**< 0.001**	0.695 (0.581, 0.832)	
Work environment	Open-pit	-	-	-	-	-	1.10
	Underground	0.324	0.073	19.89	**0.000**	1.383 (1.199, 1.595)	
Job burnout	Zero burnout (Ref.)	-	-	89.28	**< 0.001**	-	1.04
	Mild burnout	0.337	0.094	12.85	**< 0.001**	1.401 (1.165, 1.685)	
	Moderate burnout	0.784	0.101	59.76	**< 0.001**	2.190 (1.795, 2.672)	
	Severe burnout	1.809	0.286	39.90	**< 0.001**	6.102 (3.481, 10.694)	
Occupational stress	Non-occupational stress (Ref.)	-	-	-	-	-	1.04
	Occupational stress	0.379	0.071	28.81	**< 0.001**	1.462 (1.272, 1.679)	

VIF, Variance Inflation Factor. The bold values indicate the values of *P* < 0.05.

### Structural equation model of occupational stress–job burnout–psychological symptoms

**Figure 2:**
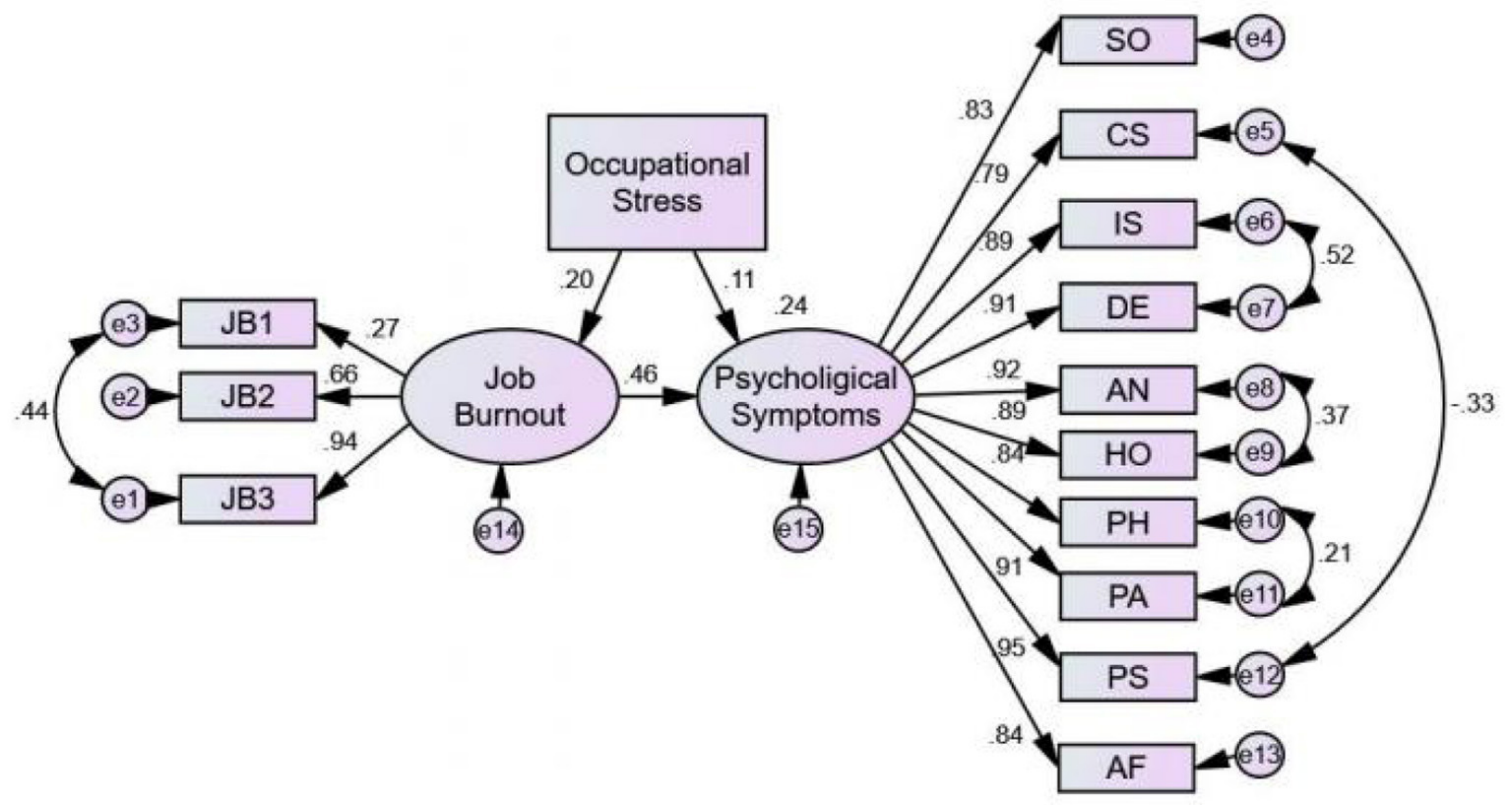
Structural equation modeling results for the relationship between occupational stress, burnout, and psychological symptoms. All coefficients in the figure are standardized and significant at the 0.05 level. JBI, emotional exhaustion; JB2, depersonalization; JB3, reduced personal accomplishment; SO, Somatization; CS, Compulsive symptoms; IS, Interpersonal sensitivity; DE, Depression.

Based on the principles of accuracy and simplicity, the model was continuously revised by using AMOS software to obtain the optimal structural equation model of occupational stress-burnout-psychological symptoms (Figure 2). The model fitted well (CFI = 0.973, GFI = 0.945, AGFI = 0.907, TLI = 0.961, RMSEA = 0.078) and the differences were significant statistically (*P* < 0.001). The results of structural equation modeling indicated that occupational stress (β = 0.11, *P* = 0.002) and job burnout (β = 0.46, *P* = 0.002) had significant positive direct effects on psychological symptoms, and occupational stress had an indirect effect on psychological symptoms through the intermediate variable job burnout, with the mediating effect of 0.20 × 0.46 = 0.092, accounting for 47.18% of the total effect.

## Discussion

This study showed that higher levels of occupational stress and burnout were associated with increased detection of positive psychological symptoms in coal miners. Poor mental health status is associated with the unsafe behaviors of coal miners and has a predictive effect on the occurrence of coal mine accidents (50). Consequently, this study provides the data support and intervention direction for coal mine management enterprises to pay attention to the psychological health of coal miners, which is also significant in improving the occupational life quality of coal miners and ensuring the safety production of coal mining enterprises.

The prevalence of psychological disorders is increasing worldwide, and psychological disorders are now a major cause of global disease burden and disability (51). The occurrence of psychological symptoms was affected by many factors, including both the individual demographic and job characteristics of the coal miners (10, 38). This study found significant differences in the positive detection rate of psychological symptoms among different coal miner characteristic groups. These differences depended on the participants' gender, educational level, marital status, shift assignment, monthly income, work environment and alcohol use (*P* < 0.05). The results of logistic regression analysis also showed that these factors were influencing factors for psychological symptoms among coal miners except for alcohol use.

The prevalence of psychological symptoms was higher among males and underground workers than among females and open-pit coal miners, which was probably related to the fact that all the underground workers in this study were males. Studies have shown that, except for healthcare occupations and occupations that necessitate long periods of standing, males (especially middle-aged males) tend to be exposed to hazardous chemicals more often, and are at a higher risk of physical harm and job stress and burnout (52). Underground mining is a highly hazardous activity that provides workers with a narrow space to move around (53). Other studies have also indicated that underground coal miners, most of whom were males, not only worked in hazardous environments away from family and friends but were also subjected to stressors associated with the work environment, and those challenging conditions increased the risk of psychological symptoms among underground coal miners (40). The prevalence of psychological symptoms was higher among miners with a college degree education or above than among those with a high school education or below. Educational levels can modulate mental health, and higher levels of education may protect people from symptoms of anxiety and depression when they are confronted with adverse events (51). However, the working environment and working characteristics of coal miners were special, and most of the participants had a low level of education. While miners with a higher education held job positions that corresponded to their educational level, miners in leadership positions reported higher levels of occupational stress and job demands, and an interaction was observed between job position and occupational stress in relation to psychological health (54), which may explain the higher incidence of psychological symptoms among coal miners with higher education levels than among those with low education levels. In this study, the prevalence of psychological symptoms was higher among those who were divorced or widowed than among those who were married. This finding was consistent with the results of numerous prior studies. Divorce, separation, or widowhood were all associated with an increased risk of alcoholism, anxiety, and depression (55). People with psychological symptoms have lower rates of marriage, and marriage may be a protective factor for mental disorders in the long run (56). A cross-sectional epidemiological survey of 28,140 psychological symptoms involving 31 provinces in China showed that individuals who were separated, widowed, or divorced were 1.87-times more likely to develop psychological symptoms than cohabiting or married individuals (57). The prevalence of psychological symptoms in this study was lower in the work shifts group (excluding night shifts) than in the day shift group. At present, the conclusions on the health effects of shifts work in occupational groups have not been unified. Most cross-sectional studies found a correlation between work shift and chronic disease, decreased fertility, insomnia disorders, and poor mental health (58, 59). However, some longitudinal studies have shown that shift workers (especially males) have better mental health than non-shift workers (60). The inconsistency of these findings may be attributed to cultural differences, and differences in the occupations and shift work trends of the study populations (2). In our study, the prevalence of psychological symptoms was higher among coal miners with a monthly income of <5,000 yuan than among those who earned ≥5,000 yuan. Studies have shown that a higher prevalence of psychological symptoms was associated with lower socioeconomic status and economic income (1), with lower-income individuals being more likely to experience psychological symptoms and poorer mental health being more prevalent among miners earning <4,000 yuan per month (14). These may be due to their inability to adapt quickly to fast-paced social changes, exposure to severe adverse life events, violence, and risk of poor physical health (61). In this study, the prevalence of psychological symptoms was higher among alcohol users than non-alcohol users. The alcohol use rate among coal miners was high, and higher rates of harmful alcohol use were more pronounced among males, younger age groups and people with psychological symptoms (62).

Among the 4,109 coal miners in the study, 3,179 reported different levels of job burnout (77.4%), the positive detection rate of psychological symptoms increased in line with higher burnout levels. Job burnout played a significant role in anxiety and depression levels in the occupational population, with statistically significant correlations between emotional exhaustion, depersonalization, anxiety, and depression (36). Multivariate results suggest that higher levels of burnout may be risk factors for psychological symptoms in coal miners. That was consistent with the finding that the risk of psychological symptoms was 2.59 times higher among workers with severe levels of burnout than workers with zero job burnout (63). The results of SEM indicated that job burnout had significantly positive and direct effects on psychological symptoms, and burnout was an intermediate variable between occupational stress and psychological symptoms. Other studies have also shown an association between psychological symptoms and job burnout in factory and mine workers, and that job burnout may also intermediate the relationship between job stress and psychological symptoms (64). Chronic high levels of job burnout can adversely affect the motivation and job satisfaction of coal miners, thereby affecting employee morale in the workforce (65). Job burnout was associated with excessive working hours (18), high job demands, and lower job control and decision-making freedom (19). Minimizing emotional exhaustion, increasing social support, and reducing occupational stressors in occupational work environments will help to prevent depression and anxiety (26).

Among the 4,109 coal miners in the study, 1,564 workers had occupational stress (38.1%), and the detection rate of psychological symptoms in the occupational stress group was higher than that in the non-occupational stress group. The multivariate results showed that occupational stress in coal miners was a risk factor for psychological symptoms, furthermore, the SEM results also indicated that occupational stress had direct and indirect effects on psychological symptoms. This finding demonstrated a close association between the occupational stress of coal miners and psychological symptoms. Studies have also reported that occupational stress was negatively correlated with mental health in occupational populations (64). Occupational stress was identified as an important factor affecting job burnout and depressive symptoms among coal miners in Xinjiang, China (24). Coal mining is characterized as a high-risk occupation, with insufficient organizational support and a lack of job security. The degree of occupational stress was positively correlated with the degree of criticality and threat in the working environment (30), which may explain the high degree of occupational stress among coal miners. The economic resources of coal miners were relatively scarce, the working environment was poor, and work-related negative emotions were strong. When the workers experienced psychological distress, they were usually not proactive in seeking help, which increased their risk of psychological symptoms (66).

## Conclusion

The psychological health of coal miners was affected by many factors, and high levels of job burnout and occupational stress were risk factors for psychological symptoms. Both occupational stress and job burnout had direct effects on psychological symptoms, and occupational stress could also have an indirect effect on coal miners' psychological symptoms through the intermediate variable of burnout. To improve the quality of the occupational lives of the miners and ensure safe production by coal mining enterprises, the relevant and responsible coal mining units should pay attention to monitoring the mental health of the coal miners, alleviate the mental burden of these workers, strive for better worker welfare standards, and strengthen the interaction between miners. Health education and occupational safety training should be carried out regularly to reduce the risk of psychological symptoms among coal miners.

### Strengths and study limitations

Our study aimed to explore the association between occupational stress, job burnout, and psychological symptoms through large sample size data and further explored their structural relationship by structural equation modeling, which complemented the research lacuna that occupational stress among coal miners in Xinjiang can affect psychological symptoms through burnout as an intermediate variable. Although our study was a cross-sectional study, we selected the study subjects strictly according to the inclusion and exclusion criteria and used a measurement questionnaire with high reliability and validity, all of which minimized bias. However, there were still some limitations in our study. First, our study was a cross-sectional epidemiological survey in the form of an electronic questionnaire, and there may have been some recall bias, but the reliability and validity of the questionnaire in this study were high, which minimized the recall bias. Second, we only demonstrated an association between job burnout, occupational stress, and psychological symptoms, but did not elaborate upon a causal relationship, our studies in the future will focus on exploring and verifying the causal relationship. Third, our study population included coal miners in Xinjiang, China, and any generalization of the findings is limited to a certain extent. Therefore, the conclusions reached need to be verified in other populations. At last, because the work type in our questionnaire was fill-in-the-blank rather than multiple-choice, we collected too much information about the work type, however, it was hard to make an accurate classification, so we failed to analyze the effect of work type on psychological symptoms, in the future study, we will pay attention to a research design with comprehensive consideration.

## Data availability statement

The original contributions presented in the study are included in the article/supplementary material, further inquiries can be directed to the corresponding author/s.

## Ethics statement

The studies involving human participants were reviewed and approved by the Medical Ethics Committee of the First Affiliated Hospital of Xinjiang Medical University (Approval number: 20170214-174). The patients/participants provided their written informed consent to participate in this study.

## Author contributions

AF and JL designed the study. TZ, XLi, and XLiu designed the electronic version of the questionnaire and conducted the investigation. TZ, XG, XLi, and XLiu carried out the data detection and formal analysis. AF and TZ were involved in writing and editing the first manuscript. All authors approved the manuscript.

## References

[1] Considine R Tynan R James C Wiggers J Lewin T Inder K. The contribution of individual, social and work characteristics to employee mental health in a coal mining industry population. PLoS ONE. (2017) 12:e0168445. 10.1371/journal.pone.0168445 28045935 PMC5207427

[2] Torquati L Mielke GI Brown WJ Burton NW Kolbe-Alexander TL. Shift work and poor mental health: a meta-analysis of longitudinal studies. Am J Public Health. (2019) 109:e13–20. 10.2105/AJPH.2019.305278 31536404 PMC6775929

[3] Lu Y Yan H Yang J Liu J. Occupational stress and mental health impact on hypertension of miners in noisy environment in Wulumuqi, China: a case-control study. BMC Public Health. (2020) 20:1675. 10.1186/s12889-020-09760-9 33167970 PMC7653708

[4] Li X Yang X Sun X Xue Q Ma X Liu J. Associations of musculoskeletal disorders with occupational stress and mental health among coal miners in Xinjiang, China: a cross-sectional study. BMC Public Health. (2021) 21:1327. 10.1186/s12889-021-11379-3 34229637 PMC8259414

[5] Bustamante-Granda BF Rodríguez-Hidalgo C Cisneros-Vidal MA Rivera-Rogel D Torres-Montesinos C. Ecuadorian journalists mental health influence on changing job desire: a cross sectional study. Int J Environ Res Public Health. (2021) 18:10139. 10.3390/ijerph181910139 34639441 PMC8508482

[6] Thommasen HV Lavanchy M Connelly I Berkowitz J Grzybowski S. Mental health, job satisfaction, and intention to relocate. Opinions of physicians in rural British Columbia. Can Fam Phys. (2001) 47:737–44. 11340754 PMC2018427

[7] Zhang J Zhang X. Chinese college students' SCL-90 scores and their relations to the college performance. Asian J Psychiatr. (2013) 6:134–40. 10.1016/j.ajp.2012.09.009 23466110

[8] Liu Y Liu J Zhu B. A study on the mental health status of underground coal mine workers in China, 2007-2014. J Health Saf Environ. (2018) 18:176–81. 10.13637/j.issn.1009-6094.2018.01.034

[9] Stansfeld SA Rasul FR Head J Singleton N. Occupation and mental health in a national UK survey. Soc Psychiatry Psychiatr Epidemiol. (2011) 46:101–10. 10.1007/s00127-009-0173-7 20033130 PMC3034883

[10] James C Rahman M Bezzina A Kelly B. Factors associated with patterns of psychological distress, alcohol use and social network among Australian mineworkers. Aust N Z J Public Health. (2020) 44:390–6. 10.1111/1753-6405.13037 32865849

[11] Nørøxe KB Pedersen AF Bro F Vedsted P. Mental well-being and job satisfaction among general practitioners: a nationwide cross-sectional survey in Denmark. BMC Fam Pract. (2018) 19:130. 10.1186/s12875-018-0809-3 30055571 PMC6064618

[12] Kinman G Clements AJ Hart J. Job demands, resources and mental health in UK prison officers. Occup Med (Lond). (2017) 67:456–60. 10.1093/occmed/kqx091 28898963

[13] Piao X Managi S. Long-term improvement of psychological well-being in the workplace: what and how. Soc Sci Med. (2022) 298:114851. 10.1016/j.socscimed.2022.114851 35272248

[14] Han S Chen H Harris J Long R. Who reports low interactive psychology status? An investigation based on Chinese Coal Miners. Int J Environ Res Public Health. (2020) 17:2853. 10.3390/ijerph18062853 32429127 PMC7277538

[15] Hori D Oi Y Ohtaki Y Andrea CS Takahashi T Shiraki N. Association between flourishing mental health and occupational stress among workers of Tsukuba Science City, Japan: a cross-sectional study. Environ Health Prev Med. (2019) 24:64. 10.1186/s12199-019-0823-7 31775617 PMC6882175

[16] Yang X Zhang B Wang L Cao L Tong R. Exploring the relationships between safety compliance, safety participation and safety outcomes: considering the moderating role of job burnout. Int J Environ Res Public Health. (2021) 18:4223. 10.3390/ijerph18084223 33923507 PMC8073523

[17] Edú-Valsania S Laguía A Moriano JA. Burnout: a review of theory and measurement. Int J Environ Res Public Health. (2022) 19:1780. 10.3390/ijerph19031780 35162802 PMC8834764

[18] Sequera-Martín M Ramos-Fuentes MI Garrido-Ardila EM Sánchez-Sánchez C. de la Torre-Risquez A, Rodríguez-Mansilla J. Prevalence of burnout syndrome and job satisfaction in music therapists in spain: a cross-sectional, descriptive study. Int J Environ Res Public Health. (2021) 18:9108. 10.3390/ijerph18179108 34501697 PMC8430565

[19] Evans S Huxley P Gately C Webber M Mears A Pajak S. Mental health, burnout and job satisfaction among mental health social workers in England and Wales. Br J Psychiatry. (2006) 188:75–80. 10.1192/bjp.188.1.75 16388074

[20] Alenezi A McAndrew S Fallon P. Burning out physical and emotional fatigue: Evaluating the effects of a programme aimed at reducing burnout among mental health nurses. Int J Ment Health Nurs. (2019) 28:1042–52. 10.1111/inm.12608 31231965

[21] Rossi A Cetrano G Pertile R Rabbi L Donisi V Grigoletti L. Burnout, compassion fatigue, and compassion satisfaction among staff in community-based mental health services. Psychiatry Res. (2012) 200:933–8. 10.1016/j.psychres.2012.07.029 22951335

[22] Queirós C Passos F Bártolo A Faria S Fonseca SM Marques AJ. Job stress, burnout and coping in police officers: relationships and psychometric properties of the organizational police stress questionnaire. Int J Environ Res Public Health. (2020) 17:6718. 10.3390/ijerph17186718 32942672 PMC7557776

[23] Salvagioni DAJ Melanda FN Mesas AE González AD Gabani FL Andrade SM. Physical, psychological and occupational consequences of job burnout: a systematic review of prospective studies. PLoS ONE. (2017) 12:e0185781. 10.1371/journal.pone.0185781 28977041 PMC5627926

[24] Yong X Gao X Zhang Z Ge H Sun X Ma X. Associations of occupational stress with job burn-out, depression and hypertension in coal miners of Xinjiang, China: a cross-sectional study. BMJ Open. (2020) 10:e036087. 10.1136/bmjopen-2019-036087 32690741 PMC7375507

[25] Denning M Goh ET Tan B Kanneganti A Almonte M Scott A. Determinants of burnout and other aspects of psychological well-being in healthcare workers during the Covid-19 pandemic: a multinational cross-sectional study. PLoS ONE. (2021) 16:e0238666. 10.1371/journal.pone.0238666 33861739 PMC8051812

[26] Chen J Li J Cao B Wang F Luo L Xu J. Mediating effects of self-efficacy, coping, burnout, and social support between job stress and mental health among young Chinese nurses. J Adv Nurs. (2020) 76:163–73. 10.1111/jan.14208 31566806

[27] Kawakami N Fujigaki Y. Reliability and validity of the Japanese Version of Job Content Questionnaire: replication and extension in computer company employees. Ind Health. (1996) 34:295–306. 10.2486/indhealth.34.295 8908841

[28] Del Pozo-Antúnez JJ Ariza-Montes A Fernández-Navarro F Molina-Sánchez H. Effect of a job demand-control-social support model on accounting professionals' health perception. Int J Environ Res Public Health. (2018) 15:2437. 10.3390/ijerph15112437 30388812 PMC6265784

[29] Rössler W. Stress, burnout, and job dissatisfaction in mental health workers. Eur Arch Psychiatry Clin Neurosci. (2012) 262 Suppl 2:S65–69. 10.1007/s00406-012-0353-4 22926058

[30] Ritchie J Basevitch I Rodenberg R Tenenbaum G. Situation criticality and basketball officials' stress levels. J Sports Sci. (2017) 35:2080–7. 10.1080/02640414.2016.1255770 27879168

[31] Ravesteijn B Kippersluis HV Doorslaer EV. The wear and tear on health: what is the role of occupation? Health Econ. (2018) 27:e69–86. 10.1002/hec.3563 28901590 PMC5849488

[32] Schiöler L Söderberg M Rosengren A Järvholm B Torén K. Psychosocial work environment and risk of ischemic stroke and coronary heart disease: a prospective longitudinal study of 75 236 construction workers. Scand J Work Environ Health. (2015) 41:280–7. 10.5271/sjweh.3491 25785576

[33] Degen L Linden K Seifried-Dübon T Werners B Grot M Rind E. Job satisfaction and chronic stress of general practitioners and their teams: baseline data of a cluster-randomised trial (IMPROVEjob). Int J Environ Res Public Health. (2021) 18:9458. 10.3390/ijerph18189458 34574383 PMC8466539

[34] Loerbroks A Cho SI Dollard MF Zou J Fischer JE Jiang Y. Associations between work stress and suicidal ideation: Individual-participant data from six cross-sectional studies. J Psychosom Res. (2016) 90:62–9. 10.1016/j.jpsychores.2016.09.008 27772561

[35] Hege A Lemke MK Apostolopoulos Y. Sönmez S. The impact of work organization, job stress, and sleep on the health behaviors and outcomes of US long-haul truck drivers health. Educ Behav. (2019) 46:626–36. 10.1177/1090198119826232 30770029

[36] Papathanasiou IV Tsaras K Kleisiaris CF Fradelos EC Tsaloglidou A Damigos D. Anxiety and depression in staff of mental units: the role of burnout. Adv Exp Med Biol. (2017) 987:185–97. 10.1007/978-3-319-57379-3_17 28971458

[37] Kang SK Kim EA. Occupational diseases in Korea. J Korean Med Sci. (2010) 25:S4–12. 10.3346/jkms.2010.25.S.S4 21258589 PMC3023359

[38] Krüger T Kraus T Kaifie A. A changing home: a cross-sectional study on environmental degradation, resettlement and psychological distress in a Western German coal-mining region. Int J Environ Res Public Health. (2022) 19:7143. 10.3390/ijerph19127143 35742391 PMC9223024

[39] Yang L Birhane GE Zhu J Geng J. Mining employees safety and the application of information technology in coal mining: review. Front Public Health. (2021) 9:709987. 10.3389/fpubh.2021.709987 34485234 PMC8416457

[40] Stewart AG. Mining is bad for health: a voyage of discovery. Environ Geochem Health. (2020) 42:1153–65. 10.1007/s10653-019-00367-7 31289975 PMC7225204

[41] Wang K Tang H Wang F Miao Y Liu D. Research on complex air leakage method to prevent coal spontaneous combustion in longwall goaf. PLoS ONE. (2019) 14:e0213101. 10.1371/journal.pone.0213101 30822333 PMC6396930

[42] Sun X. Study on the Relationship Between Mental Health and Musculoskeletal Disease and Gene Environment Interaction Of Coal Miners. Ürümqi: Xinjiang Medical University. (2020).

[43] Li R Gao X Liu B Ge H Ning L Zhao J. Prospective cohort study to elucidate the correlation between occupational stress and hypertension risk in oil workers from Kelamayi city in the xinjiang uygur autonomous region of China. Int J Environ Res Public Health. (2017) 14:1. 10.3390/ijerph14010001 28025517 PMC5295252

[44] Li Y Wu M. A study on the structure of job burnout. Psychol Sci. (2005) 25:70–73. 10.16719/j.cnki.1671-6981.2005.02.051

[45] Dai J Yu H Wu J Fu H. Stress assessment model based on a simple job stress questionnaire in Chinese. Fudan Univ J Med Sci. (2007) 34:656–61. 10.3969/j.issn.1672-8467.2007.05.005

[46] Karasek R Baker D Marxer F Ahlbom A Theorell T. Job decision latitude, job demands, and cardiovascular disease: a prospective study of Swedish men. Am J Public Health. (1981) 71:694–705. 10.2105/AJPH.71.7.694 7246835 PMC1619770

[47] Chang HY Lo CL Hung YY. Development and validation of traditional & complementary medicine (TCM) scales for nurses: using structural equation modelling (SEM). BMC Complement Altern Med. (2019) 19:321. 10.1186/s12906-019-2733-z 31752832 PMC6868815

[48] Graham MA Eloff I. Comparing mental health, wellbeing and flourishing in undergraduate students pre- and during the COVID-19 pandemic. Int J Environ Res Public Health. (2022) 19:7438. 10.3390/ijerph19127438 35742686 PMC9224479

[49] Enders CK Mansolf M. Assessing the fit of structural equation models with multiply imputed data. Psychol Methods. (2018) 23:76–93. 10.1037/met0000102 27893216

[50] Qin K Jia Z Lu T Liu S Lan J You X. The role of work engagement in the association between psychological capital and safety citizenship behavior in coal miners: a mediation analysis. Int J Environ Res Public Health. (2021) 18:9303. 10.3390/ijerph18179303 34501896 PMC8431525

[51] Di Novi C Leporatti L Montefiori M. The role of education in psychological response to adverse health shocks. Health Policy. (2021) 125:643–50. 10.1016/j.healthpol.2021.02.006 33674133

[52] Biswas A Harbin S Irvin E Johnston H Begum M Tiong M. Sex and gender differences in occupational hazard exposures: a scoping review of the recent literature. Curr Environ Health Rep. (2021) 8:267–80. 10.1007/s40572-021-00330-8 34839446 PMC8627292

[53] Ahmad SR Akram MM. Thygerson SM, Ali Nadeem F, Khan WU. Cross-sectional survey of musculoskeletal disorders in workers practicing traditional methods of underground coal mining. Int J Environ Res Public Health. (2020) 17:2566. 10.3390/ijerph17072566 32283589 PMC7177932

[54] Siddiqui A Jia H He Y Li Y Zhen S Chiang S. Correlation of job stress and self-control through various dimensions in Beijing hospital staff. J Affect Disord. (2021) 294:916–23. 10.1016/j.jad.2021.07.094 34375220

[55] Mougharbel F Sampasa-Kanyinga H Heidinger B Corace K Hamilton HA Goldfield GS. Psychological and demographic determinants of substance use and mental health during the COVID-19 pandemic. Front Public Health. (2021) 9:680028. 10.3389/fpubh.2021.680028 34249844 PMC8270652

[56] Aggarwal S Grover S Chakrabarti S. A comparative study evaluating the marital and sexual functioning in patients with schizophrenia and depressive disorders. Asian J Psychiatr. (2019) 39:128–34. 10.1016/j.ajp.2018.12.021 30616160

[57] Lu J Xu X Huang Y Li T Ma C Xu G. Prevalence of depressive disorders and treatment in China: a cross-sectional epidemiological study. Lancet Psychiatry. (2021) 8:981–90. 10.1016/S2215-0366(21)00251-0 34559991

[58] Deng N Kohn TP Lipshultz LI Pastuszak AW. The relationship between shift work and men's health. Sex Med Rev. (2018) 6:446–56. 10.1016/j.sxmr.2017.11.009 29371140

[59] Rosa D Terzoni S Dellafiore F Destrebecq A. Systematic review of shift work and nurses' health. Occup Med (Lond). (2019) 69:237–43. 10.1093/occmed/kqz063 31132107

[60] Nabe-Nielsen K Garde AH Albertsen K Diderichsen F. The moderating effect of work-time influence on the effect of shift work: a prospective cohort study. Int Arch Occup Environ Health. (2011) 84:551–9. 10.1007/s00420-010-0592-5 21069537

[61] Patel V Kleinman A. Poverty and common mental disorders in developing countries. Bull World Health Organ. (2003) 81:609–15. 10.1037/e538812013-019 14576893 PMC2572527

[62] James CL Tynan RJ Bezzina AT Rahman MM Kelly BJ. Alcohol consumption in the australian mining industry: the role of workplace, social, and individual factors. Workplace Health Saf. (2021) 69:423–34. 10.1177/21650799211005768 33896275

[63] Lu Y Zhang Z Gao S Yan H Zhang L Liu J. Association of occupational burnout and occupational exposure factors on mental health among factory workers and miners: a propensity score analysis. Int Arch Occup Environ Health. (2021) 94:441–50. 10.1007/s00420-020-01587-6 33108547

[64] Moreno Fortes A Tian L Huebner ES. Occupational stress and employees complete mental health: a cross-cultural empirical study. Int J Environ Res Public Health. (2020) 17:10. 10.3390/ijerph17103629 32455763 PMC7277686

[65] Onyett S. Revisiting job satisfaction and burnout in community mental health teams. J Ment Health. (2011) 20:198–209. 10.3109/09638237.2011.556170 21406021

[66] Lundberg U. Stress responses in low-status jobs and their relationship to health risks: musculoskeletal disorders. Ann N Y Acad Sci. (1999) 896:162–72. 10.1111/j.1749-6632.1999.tb08113.x 10681896

